# Association of Age With Risk of Adverse Pathological Findings at Radical Prostatectomy in Men With Gleason Score 6 Prostate Cancer

**DOI:** 10.1001/jamanetworkopen.2020.2041

**Published:** 2020-04-02

**Authors:** Daniel W. Kim, Ming-Hui Chen, Hartwig Huland, Markus Graefen, Derya Tilki, Anthony V. D’Amico

**Affiliations:** 1 Department of Radiation Oncology, Dana-Farber Cancer Institute, Brigham and Women’s Hospital, Boston, Massachusetts; 2 Department of Statistics, University of Connecticut, Storrs; 3 Martini-Klinik Prostate Cancer Center, University Hospital Hamburg-Eppendorf, Hamburg, Germany; 4 Department of Urology, University Hospital Hamburg-Eppendorf, Hamburg, Germany

## Abstract

This cohort study examines the association of older age with risk of adverse pathological findings at radical prostatectomy in men with biopsy-confirmed Gleason score 6 prostate cancer.

## Introduction

The preferred treatment for men with low-risk prostate cancer, particularly men older than 65 years, is active surveillance.^1^ However, advancing age is associated with upgrading and upstaging at radical prostatectomy. Several factors, including increasing prostate-specific antigen (PSA) level, clinical tumor category, percentage of positive biopsy results, and PSA density, have been noted to be associated with clinically significant prostate cancer at radical prostatectomy.^2^ Until now, to our knowledge, no study has incorporated these factors within predefined age strata to ascertain whether a cohort of patients at high risk can be identified for whom additional evaluation and possible treatment is indicated rather than active surveillance.

## Methods

This prospective cohort study included men with Gleason score 6 prostate cancer who were treated with radical prostatectomy from February 28, 1992, to February 15, 2016, at the Martini-Klinik Prostate Cancer Center of the University Hospital Hamburg-Eppendorf in Hamburg, Germany. This study was approved, including waivers of consent owing to deidentified data and a no-risk protocol, by the Ethik-Kommission der Ärztekamme institutional review board in Hamburg, Germany. This study was reported following the Consolidated Standards of Reporting Trials (CONSORT) reporting guideline.

We investigated whether men older than 65 years had increased odds of adverse pathological findings at radical prostatectomy, defined as TNM category pT3/T4 or R1 or Gleason score 8, 9, or 10, compared with men 65 years and younger. We dichotomized age at 65 years, a commonly used cutoff, to enable clinical utility of the results. Descriptive statistics were used to compare the proportion of clinical characteristics at presentation among men older than 65 years vs 65 years and younger using a Wilcoxon rank sum test for continuous covariates and the Maental-Haenszal χ^2^ test for categorical covariates.^3^ Univariable and multivariable logistic regressions were used to calculate unadjusted and adjusted odds ratios (ORs) of adverse pathological findings at radical prostatectomy in men older than 65 years vs men 65 years and younger, adjusting for pre–radical prostatectomy PSA level, clinical tumor category, year of diagnosis, percentage of positive biopsy results, and PSA density.^3^ SAS statistical software version 9.4 (SAS Institute) was used for all statistical analysis. *P* values were 2-sided, and statistical significance was set at *P* < .05. Data were analyzed on May 24, 2019.

## Results

A total of 3191 men (median [interquartile range] age, 62 [32-77] years) were included in the study. The median (interquartile range) PSA level was 6.74 (0.14-187.00) ng/mL (to convert to micrograms per liter, multiply by 1), and 2809 men (88.3%) had T category 1c prostate cancer. Men older than 65 years, compared with men 65 years and younger, had a significantly lower median (interquartile range) percentage of positive biopsy results (16.7% [12.5%-33.3%] vs 20.0% [12.5%-37.5%]; *P* = .01) and PSA density (0.13 [0.09-0.19] ng/mL vs 0.15 [0.11-0.23] ng/mL; *P* < .001) (Table 1). While increasing percentage of positive biopsy results (adjusted OR per 1-unit increase, 1.02; 95% CI, 1.01-1.02; *P* < .001) and PSA density (adjusted OR per 1-unit increase, 4.28; 95% CI, 1.66-11.01; *P* = .003) were significantly associated with increased odds of adverse pathological findings at radical prostatectomy (Table 2), men older than 65 years had higher odds of adverse pathological findings at radical prostatectomy compared with men 65 years and younger (adjusted OR, 1.28, 95% CI, 1.00-1.62; *P* = .048).

**Table 1.** Baseline Distribution of Factors Associated with Adverse Pathological Findings at Radical Prostatectomy Stratified by Age

Characteristic	Median (IQR)		*P* value
	Men aged >65 y (n = 1075)	Men aged ≤65 y (n = 2116)	
PSA, ng/mL	6.90 (5.00-10.00)	6.65 (4.92-9.74)	.17
T category, No. (%)			
≥2a	126 (11.7)	256 (12.1)	.76
1c	949 (88.3)	1860 (87.9)	
Year of diagnosis	2008 (2004-2011)	2008 (2003-2011)	.92
Positive biopsy results, %	16.7 (12.5-33.3)	20.0 (12.5-37.5)	.01
PSA density, ng/mL/cc	0.13 (0.09-0.19)	0.15 (0.11-0.23)	<.001

Abbreviations: IQR, interquartile range; PSA, prostate-specific antigen; T, tumor.

SI conversion factor: To convert PSA level to micrograms per liter, multiple by 1.

**Table 2.** Unadjusted and Adjusted Odds Ratio of Adverse Pathological Findings at Radical Prostatectomy for Each Clinical Characteristic

Characteristic	Men, No.	Events, No.^a^	Univariable analysis		Multivariable analysis	
			Unadjusted OR (95% CI)	*P* value	Adjusted OR (95% CI)	*P* value
Age	3191	377	1.01 (1.00-1.03)^b^	.09	1.02 (1.00-1.04)^b^	.07
>65 y	1075	133	1.08 (0.87-1.36)	.49	1.28 (1.00-1.62)	.048
≤65 y	2116	244	1 [Reference]	NA	1 [Reference]	NA
PSA level	3191	377	1.02 (1.01-1.036)^b^	.004	0.99 (0.97-1.01)^b^	.95
T category						
≥2a	382	84	2.42 (1.85-3.17)	<.001	1.26 (0.93-1.71)	.13
1c	2809	293	1 [Reference]	NA	1 [Reference]	NA
Year of diagnosis	3191	377	0.87 (0.85-0.89)^b^	<.001	0.89 (0.87-0.91)^b^	<.001
Positive biopsy results, %	3191	377	1.03 (1.02-1.039)^b^	<.001	1.02 (1.01-1.02)^b^	<.001
PSA density	3191	377	3.23 (1.95-5.35)^b^	<.001	4.28 (1.66-11.01)^b^	.003

Abbreviations: NA, not applicable; OR, odds ratio; PSA, prostate-specific antigen; T, tumor.

^a^ Includes adverse pathological findings at prostatectomy, including T3/T4, R1, and/or Gleason score 8, 9, or 10.

^b^ Per 1-unit increase.

## Discussion

This cohort study found that being older than 65 years was associated with adverse pathological findings at radical prostatectomy. Specifically, if being older than 65 years was not associated with increased risk, one would have expected men older than 65 years to have a lower risk of having adverse pathological findings given the more favorable percentage of positive biopsy results and PSA density levels.

Possible explanations for the association of advancing age with risk of adverse pathological features include sampling error and undergrading owing to benign prostatic hyperplasia that occurs normally with advancing age. Another possible explanation is that most men undergo andropause starting at approximately age 40 years continuing to the end of life.^4^ Therefore, older men are more likely to have lower testosterone levels at prostate cancer diagnosis compared with younger men, and it is known that prostate cancer in men who are hypogonadal can be more aggressive compared with prostate cancer in men who are eugonadal.^5^ This study has some limitations, such as that we chose age 65 years as our cutoff for age, as it is commonly used in prostate cancer studies when distinguishing men of older vs younger age; however, life expectancy using a validated metric, such as the Adult Comorbidity Evaluation-27,^6^ may be preferred rather than a specific age cutoff.

These findings suggest that men older than 65 years with biopsy-confirmed Gleason score 6 prostate cancer may benefit from additional testing, such as multiparametric magnetic resonance imaging and targeted biopsy before proceeding with active surveillance. If higher grade or stage disease is detected, this information could be used to guide the use and duration of androgen deprivation therapy in men considering radiotherapy.
